# Abnormal skeletal muscle blood flow, contractile mechanics and fibre morphology in a rat model of obese‐HFpEF

**DOI:** 10.1113/JP280899

**Published:** 2021-01-04

**Authors:** Ever Espino‐Gonzalez, Peter G. Tickle, Alan P. Benson, Roger W. P. Kissane, Graham N. Askew, Stuart Egginton, T. Scott Bowen

**Affiliations:** ^1^ School of Biomedical Sciences Faculty of Biological Sciences University of Leeds Leeds UK; ^2^ Department of Musculoskeletal & Ageing Science University of Liverpool Liverpool UK

**Keywords:** blood flow, diaphragm, heart failure, HFpEF, muscle atrophy, muscle contraction, skeletal muscle

## Abstract

**Key points:**

- Heart failure is characterised by limb and respiratory muscle impairments that limit functional capacity and quality of life. However, compared with heart failure with reduced ejection fraction (HFrEF), skeletal muscle alterations induced by heart failure with preserved ejection fraction (HFpEF) remain poorly explored.
- Here we report that obese‐HFpEF induces multiple skeletal muscle alterations in the rat hindlimb, including impaired muscle mechanics related to shortening velocity, fibre atrophy, capillary loss, and an impaired blood flow response to contractions that implies a perfusive oxygen delivery limitation.
- We also demonstrate that obese‐HFpEF is characterised by diaphragmatic alterations similar to those caused by denervation – atrophy in Type IIb/IIx (fast/glycolytic) fibres and hypertrophy in Type I (slow/oxidative) fibres.
- These findings extend current knowledge in HFpEF skeletal muscle physiology, potentially underlying exercise intolerance, which may facilitate future therapeutic approaches.

**Abstract:**

Peripheral skeletal muscle and vascular alterations induced by heart failure with preserved ejection fraction (HFpEF) remain poorly identified, with limited therapeutic targets. This study used a cardiometabolic obese‐HFpEF rat model to comprehensively phenotype skeletal muscle mechanics, blood flow, microvasculature and fibre atrophy. Lean (*n* = 8) and obese‐HFpEF (*n* = 8) ZSF1 rats were compared. Skeletal muscles (soleus and diaphragm) were assessed for *in vitro* contractility (isometric and isotonic properties) alongside indices of fibre‐type cross‐sectional area, myosin isoform, and capillarity, and estimated muscle PO_2_. *In situ* extensor digitorum longus (EDL) contractility and femoral blood flow were assessed. HFpEF soleus demonstrated lower absolute maximal force by 22%, fibre atrophy by 24%, a fibre‐type shift from I to IIa, and a 17% lower capillary‐to‐fibre ratio despite increased capillary density (all *P* < 0.05) with preserved muscle PO_2_ (*P = *0.115) and isometric specific force (*P* > 0.05). Soleus isotonic properties (shortening velocity and power) were impaired by up to 17 and 22%, respectively (*P* < 0.05), while the magnitude of the exercise hyperaemia was attenuated by 73% (*P = *0.012) in line with higher muscle fatigue by 26% (*P* = 0.079). Diaphragm alterations (*P* < 0.05) included Type IIx fibre atrophy despite Type I/IIa fibre hypertrophy, with increased indices of capillarity alongside preserved contractile properties during isometric, isotonic, and cyclical contractions. In conclusion, obese‐HFpEF rats demonstrated blunted skeletal muscle blood flow during contractions in parallel to microvascular structural remodelling, fibre atrophy, and isotonic contractile dysfunction in the locomotor muscles. In contrast, diaphragm phenotype remained well preserved. This study identifies numerous muscle‐specific impairments that could exacerbate exercise intolerance in obese‐HFpEF.

## Key points

- Heart failure is characterised by limb and respiratory muscle impairments that limit functional capacity and quality of life. However, compared with heart failure with reduced ejection fraction (HFrEF), skeletal muscle alterations induced by heart failure with preserved ejection fraction (HFpEF) remain poorly explored.
- Here we report that obese‐HFpEF induces multiple skeletal muscle alterations in the rat hindlimb, including impaired muscle mechanics related to shortening velocity, fibre atrophy, capillary loss, and an impaired blood flow response to contractions that implies a perfusive oxygen delivery limitation.
- We also demonstrate that obese‐HFpEF is characterised by diaphragmatic alterations similar to those caused by denervation – atrophy in Type IIb/IIx (fast/glycolytic) fibres and hypertrophy in Type I (slow/oxidative) fibres.
- These findings extend current knowledge in HFpEF skeletal muscle physiology, potentially underlying exercise intolerance, which may facilitate future therapeutic approaches.

## Introduction

Increasing prevalence of heart failure with preserved ejection fraction (HFpEF), in the absence of recognised pharmaceutical treatments, presents one of the biggest challenges to modern cardiology (Butler *et al*. 2014; Sharma & Kass, 2014; Fukuta *et al*. 2016). While the primary pathology of HFpEF is of cardiac origin, there is a poor correlation between heart dysfunction and the main symptom of exercise intolerance (Haykowsky & Kitzman, 2014), and many clinical trials have shown cardiac‐orientated drugs are not associated with beneficial outcomes (Shah *et al*. 2016). Recent investigations, therefore, have suggested non‐cardiac ‘peripheral’ factors as major mechanisms limiting functional capacity and quality of life in patients with HFpEF, with skeletal muscle abnormalities receiving much attention (Adams *et al*. 2017; Poole *et al*. 2018; Zamani *et al*. 2020). For example, animal and human studies have shown that HFpEF is associated with various skeletal muscle impairments that are closely associated with exercise intolerance and lower quality of life, including lower skeletal muscle mass and strength (Bekfani *et al*. 2016), generalised fibre atrophy (Bowen *et al*. 2018), fat infiltration (Haykowsky *et al*. 2014; Zamani *et al*. 2020), reduced global capillary‐to‐fibre ratio (Kitzman *et al*. 2014; Bowen *et al*. 2018), reduced mitochondrial function and content (Bowen *et al*. 2015; Molina *et al*. 2016; Bowen *et al*. 2017b), disrupted high‐energy phosphate metabolism (Bhella *et al*. 2011b; Weiss *et al*. 2017), and impaired O_2_ extraction (Dhakal *et al*. 2015; Houstis *et al*. 2018; Zamani *et al*. 2020).

Despite recent progress, our current understanding of the skeletal muscle pathophysiology in HFpEF at both the structural and functional level is in its infancy, with only limited and often conflicting experimental data available (Poole *et al*. 2018). For example, it remains controversial whether leg muscle arterial blood flow (i.e. perfusive O_2_ transport) is impaired during exercise in HFpEF due to a lack of direct measurements (Hundley *et al*. 2007; Haykowsky *et al*. 2013; Lee *et al*. 2016a; Weavil *et al*. 2020), with key studies dependent upon systemic blood sampling reporting contrasting findings regarding whether a perfusive or diffusive O_2_ transport limitation impairs muscle O_2_ extraction and thus exercise intolerance in HFpEF (Dhakal *et al*. 2015; Houstis *et al*. 2018; Zamani *et al*. 2020). As such, there remains a lack of clarity on whether functional indices related to leg blood flow and perfusive O_2_ delivery are constrained in HFpEF. Likewise, we also have a limited understanding of the muscle and microvascular structural phenotype that occurs with HFpEF. For example, while studies have shown that a global fibre atrophy is present alongside a loss of capillaries‐per‐fibre (Kitzman *et al*. 2014; Bowen *et al*. 2015, 2017b, 2018; Schauer *et al*. 2020), the degree of atrophy and capillary rarefaction quantified locally across all fibre isoforms which can be further harnessed to provide novel estimates of muscle PO_2_ remains undefined. Furthermore, a knowledge gap still exists in relation to potential sites of skeletal muscle dysfunction in HFpEF that include the contribution of neuromuscular transmission *vs*. excitation–contraction failure and/or the impact on more physiologically relevant mechanical measures (i.e. shortening velocity and power), with previous studies performed using only *in vitro* isometric contractions under direct muscle stimulation (Bowen *et al*. 2015, 2017b, 2018; Schauer *et al*. 2020).

Beyond this, the majority of experimental work has been directed towards characterising the locomotor muscles despite key evidence showing that respiratory muscle dysfunction is linked to exercise intolerance in HFpEF, as shown by non‐invasive patient measures (Lavietes *et al*. 2004; Yamada *et al*. 2016) and direct diaphragm contractility measures in experimental models (Bowen *et al*. 2015, 2017b). Similar to limb muscle, however, detailed quantification of diaphragm fibre‐type morphology, capillarity, PO_2_, and clinically relevant functional measurements during cyclical length changes (i.e. as occurs during breathing) remain largely undefined in HFpEF and are unlikely to follow a similar response to the locomotor muscles.

The present study, therefore, aimed to provide a more comprehensive assessment of the skeletal muscle phenotype in HFpEF, by applying *in vitro*, *in situ* and *in silico* approaches to a validated obese cardiometabolic rat model, where *ex vivo* magnetic resonance imaging was used to characterise the degree of cardiac remodelling. Specifically, using hindlimb (soleus/EDL) and respiratory (diaphragm) muscle, we performed global and local fibre‐type specific phenotyping of cross‐sectional area, isoform, and capillarity alongside estimated muscle PO_2_. In parallel, we also directly assessed key functional measures during rest and contractions, including hindlimb blood flow as well as neural‐ and direct‐muscle stimulated contractile mechanics. We reasoned that a better understanding of the skeletal muscle phenotype in HFpEF across multiple‐system levels would provide important insights for better understanding the pathophysiology of exercise intolerance in this disease and help direct future patient experiments and therapeutic development in this field.

## Methods

### Ethical approval

All procedures and experiments were performed in accordance with the UK Scientific Procedures (Animals) Act 1986 and local approval was given by the University of Leeds Animal Welfare and Ethical Review Committee. All work conforms to the ethical requirements outlined by *The Journal of Physiology* (Grundy, 2015).

### Animals

Twenty‐week‐old male obese (*n* = 8) and lean (*n* = 8) diabetic Zucker fatty/spontaneously hypertensive heart failure F1 hybrid (ZSF1) rats (Charles River Laboratories) were used in this study. While both lean and obese ZSF1 rats inherit the hypertension gene, only the obese ZSF1 rats inherit a mutation in the leptin receptor gene (*Lepr^fa^Lepr^cp^/Crl*) that drives weight gain and the metabolic impairments associated with typical signs of HFpEF developing as early as 10 weeks of age (Schauer *et al*. 2020) and well established after 20 weeks (Leite *et al*. 2015; Franssen *et al*. 2016; van Dijk *et al*. 2016; Bowen *et al*. 2017b). Lean ZSF1 rats served as controls. All rats were maintained in a 12 h light/dark cycle, with standard chow diets (RM1 chow, SDS) and water provided *ad libitum*.

### Cardiometabolic function

Cardiometabolic impairments were confirmed by measures of body mass, mean arterial pressure (via an implanted carotid catheter (PP10)) with a blood pressure transducer (BP transducer, AD Instruments, UK) and blood glucose levels (via a commercial blood glucose meter (FreeStyle Mini Meter), while hearts were perfused and immersion fixed *ex vivo* using low osmolality Karnovsky's fixative and subsequently imaged using a diffusion‐weighted fast spin echo sequence at a resolution of 120 μm isotropic for cardiac phenotyping (Teh *et al*. 2016). These data were used to calculate mean thicknesses of the left and right ventricular free walls and the septum, for tissue located in the middle third of the distance between the base and apex of the appropriate ventricular cavity. Myocyte helix angles (quantifying myocyte inclination with respect to the short axis of the heart) were extracted from regions in the left and right ventricular free walls and the septum as previously described (Benson *et al*. 2011); myocyte disarray in these regions was quantified using the *R* ^2^ of a 5th order polynomial fit to the helix angles plotted as a function of transmural distance (Benson *et al*. 2008). All DT‐MRI analyses were carried out using in‐house software.

### *In situ* muscle performance and femoral artery blood flow

*In situ* measurements of muscle function and blood flow were made under surgical anaesthesia, which was induced with isoflurane (4% in 100% oxygen) and maintained throughout experiments by constant syringe pump infusion (30–35 mg kg^−1^ h^−1^) of Alfaxalone (Jurox, Crawley, UK) delivered via an implanted jugular vein catheter. *In situ* functional assessment of muscle performance was determined as previously described (Egginton & Hudlicka, 1999; Tickle *et al*. 2020). In brief, extensor digitorum longus (EDL) isometric twitch force was recorded via a lever arm force transducer (305B‐LR: Aurora Scientific, Aurora, ON, Canada) following surgical extirpation of the overlying synergist tibialis anterior muscles. Electrical stimulation of the EDL (0.3 ms pulse width) was accomplished via electrodes placed adjacent to the popliteal nerve (Hudlická *et al*. 1977), with initial electrical pulses (1 Hz) delivered to determine optimal muscle length and supramaximal current delivery. Simultaneous measurement of bilateral blood flow was facilitated by placement of perivascular flow probes (0.7PSB; Transonic, Ithaca, NY, USA) on the proximal portion of the femoral artery, adjacent to the *profunda femoris* bifurcation (Tickle *et al*. 2020). Quantification of resting and end‐stimulation blood flows enabled determination of the functional hyperaemia recruited during stimulation. Blood flow data are provided (ml min^−1^) and after normalisation for blood pressure variation, vascular conductance (ml min^−1 ^mm Hg^−1^). All data were recorded via PowerLab and LabChart software (AD Instruments, UK).

EDL twitch and maximal tetanic force, as well as fatigue resistance, were also assessed. Fatigue resistance was quantified by monitoring isometric force throughout a period of continuous 10 Hz stimulation for 3 min. A fatigue index was then calculated as: (end‐stimulation twitch tension/peak twitch tension) x 100. An average of five consecutive twitches was used to quantify end stimulation and peak EDL tension. Differences in the magnitude of the absolute tetanic force generated between groups were taken into account by employing a second bout of fatigue stimulation, such that absolute forces in HFpEF were initially similar to those attained in controls (i.e. matched initial force) (Ferreira *et al*. 2010). This protocol is relevant for clinical translation of muscle fatigue, where daily tasks in patients are often dependent upon the absolute rather than relative force being sustained that may involve an increase in firing frequency of motor neurons to achieve task completion (Weavil *et al*. 2020). Thus, by adjusting the stimulation frequency in HFpEF rats to around 25 Hz, tetanic force was increased and matched to the level recorded in the lean group, with fatigue allowed to proceed over 3 min. In addition, tetanic force production was quantified by 200 Hz stimulation (200 ms duration) after a minimum of 10 min recovery from fatigue, as determined by restoration of pre‐fatigue resting blood flow. All protocols were performed in exactly the same order for each rat, thus minimising any effects of methodological variation. Force is presented in absolute units (g) and normalised to wet mass (g mg^−1^).

### *In vitro* functional assessment

Immediately following killing, the soleus and diaphragm were excised and prepared in a Krebs–Henseleit solution (117 NaCl, 4.7 KCl, 1.2 MgSO_4_, 1.2 KH_2_PO_4_, 24.8 NaHCO_3_, 2.5 CaCl_2_, 11.1 glucose; in mmol l^−1^) at 4°C equilibrated with 95% O_2_/5% CO_2_. For the soleus, silk sutures (4.0) attached to tendons at either end were used to suspend the muscle vertically in a buffer‐filled organ bath between a hook and a length‐controlled lever system (305C, Aurora Scientific, Aurora, Canada). *In vitro* field stimulation using platinum electrodes was provided via a high power bipolar stimulator (701C, Aurora Scientific) outputting supramaximal current (700 mA; 1 s train duration; 0.25 ms pulse width). After optimal contractile length (*L_0_*) was determined, the muscle was thermoequilibrated in a Krebs–Henseleit solution for 15 min at ∼21°C (Bowen et al., 2017a). For the diaphragm, a bundle of muscle fascicles (∼2–3 mm wide) was removed from the medial section of the left costal diaphragm leaving two ribs and a section of the central tendon intact. The muscle bundle was transferred to a flow‐through muscle chamber and anchored between a base and ergometer (series 300B‐LR, Aurora Scientific Inc.) and stimulated through parallel platinum electrodes using a stimulus isolation unit (0.2 ms pulse width; UISO model 236, Hugo Sachs Elektronik). After *L_0_* was determined, the diaphragm bundle was thermoequilibrated in a Krebs–Henseleit solution for at least 15 min at 37°C. Each muscle was circulated with oxygenated (95% O_2_ /5% CO_2_) Krebs–Henseleit solution throughout each experiment.

The soleus underwent two protocols: isometric force–frequency and isotonic force–velocity. The force–frequency relationship was determined in response to pulses at 1, 15, 30, 50, 80, 120 and 150 Hz, with 1 min of recovery between contractions. After a 5 min period in which muscle length was measured using digital callipers, the soleus was subjected to a series of afterloaded‐isotonic contractions to determine the force–velocity relationship, where the muscle was allowed to shorten against external loads (80 – ∼5% of the maximal tetanic force; each separated by 1 min for the soleus or 5 min for the diaphragm) after being stimulated at 150 Hz for 300 ms. Shortening velocity was determined 10 ms after the first change in length and on the linear section of the transient (605A DMA software, Aurora Scientific). For the diaphragm, maximal isometric twitch and tetanic (250 ms train at 150 Hz), isotonic force–velocity (as above), and work loop protocols were performed. Muscle performance assessed using the work loop technique included simulating performance *in vivo*, by subjecting the muscle to cyclical length changes and phasic stimulation (Josephson, 1985). A sinusoidal length change at a range of cycle frequencies (1–15 Hz) and strain amplitude of 0.065 *L*_0_ was imposed on the muscle and, for each cycle frequency, the timing and duration of stimulation were optimised to maximise net work. Isometric tetanic contractions and cyclical contractions at 5 Hz were performed periodically to monitor any decline in the preparation, assessed by expressing isometric stress (isotonic contractions) and net work relative to maximal values. A linear decline in performance was assumed in correcting data for preparation decline. A period of 5 min was allowed following isotonic and work loop contractions for recovery. To assess the muscle's ability to sustain work, a fatigue test was carried out by subjecting the muscle to a series of cyclical contractions (cycle frequency 2 Hz, strain amplitude 0.065 *L*_0_, phase −20 ms relative to peak length, 210 ms stimulation duration). Custom‐written software was used to control muscle length and stimulation and to acquire length and force data (CEC Testpoint version 7) via a D/A data acquisition card (DAS1802AO, Keithley Instruments). Data were acquired at a sample frequency of 10 kHz (isometric and isotonic) or 1000 x cycle frequency (work loops).

At the end of each experiment, the muscle was blotted on paper tissue and wet mass recorded. Force (N) was normalised to muscle cross‐sectional area (CSA; cm^2^) after dividing muscle mass (g) by the product of *L_0_* (cm) and estimated muscle density (1.06 g/cm^3^) to allow specific force (i.e. stress) in N/cm^2^ to be calculated (Close, 1972). Shortening velocity was normalised to optimal muscle length (in *L_0_*/s), while power was calculated as the product of shortening velocity and force normalised to muscle mass (in W kg^−1^). Twitch properties (i.e. peak force; time‐to‐peak tension; half relaxation time) as well as maximal isometric tetanic force (i.e.$\;P_{0}$) were calculated. A hyperbolic‐linear relationship was fit to the force–velocity data to determine the maximum shortening velocity ($V_{max}$), peak isotonic power (${\dot{W}}_{max}$), and the power ratio (${\dot{W}}_{max}/\left(P_{0}\times V_{max}\right)$; i.e., a measure of the curvature of the force–velocity relationship (Marsh and Bennet, 1986).

### Histological analysis

Mid‐portions of the right costal diaphragm and left soleus muscle were mounted in optimal cutting temperature embedding medium (Thermo Scientific, Loughborough, UK), frozen in liquid nitrogen‐cooled isopentane and stored at −80°C. To identify muscle fibre types, sections (10 μm thick) were fixed for 2 min in 2% paraformaldehyde, washed in phosphate‐buffered saline (PBS; P4417, Sigma‐Aldrich, St Louis, MO) and blocked for 10 min in 1% bovine serum albumin (A6003, Sigma‐Aldrich, St Louis, MO). Sections were then incubated for 60 min with monoclonal‐myosin heavy chain antibodies BA‐D5 (IgG2B, 1:1000) and SC‐71 (IgG1, 1:500) for Type I (oxidative) and Type IIa (fast oxidative, glycolytic) fibres, respectively (Developmental Studies Hybridoma Bank, Iowa City, IA, USA). The remaining unstained fibres were considered to be Type IIb/IIx, as previously described (Kissane *et al*. 2018). After washing in PBS, sections were incubated for 60 min with secondary antibodies Alexa Fluor 555 (conjugated goat anti‐mouse IgG, 1:1000, A‐21422, Thermo Fisher Scientific, Waltham, MA) and Alexa Fluor 488 (conjugated rabbit anti‐mouse IgG, 1:1000, A11059, Thermo Fisher Scientific, Waltham, MA). Muscle fibre boundaries were labelled with a rabbit anti‐laminin antibody (1:200; L9393, Sigma‐Aldrich, St Louis, MO), an extracellular matrix glycoprotein within the basement membrane. Finally, capillaries were stained with a carbohydrate‐binding protein (lectin) specific to rodent endothelial cells, *Griffonia simplicifolia* lectin I (Vector Labs, Peterborough, UK; FL‐1101). Slides were then imaged at magnifications of x10 (soleus) and x20 (diaphragm) using the Nikon Eclipse E600 (Nikon, Tokyo, Japan) optical microscope attached to a digital camera (QIMAGING, MicroPublisher 5.0 RTV, Surrey, BC, Canada). Subsequent image analysis with the stand‐alone graphic user interface, DTect, and a MATLAB‐based oxygen transport modeller (The MathWorks, Cambridge, United Kingdom; (Al‐Shammari *et al*. 2019)) enabled calculation of fibre type‐specific cross‐sectional area (FCSA), capillary‐to‐fibre (C:F) ratio, capillary density (CD), capillary domain area (CDA), local capillary‐to‐fibre ratio (LCFR), local capillary density (LCD) and estimated tissue oxygen tension (PO_2_). Multiple regions of interest of each muscle (three for the diaphragm and two for the soleus) were randomly assigned to establish an unbiased counting frame, taking into account the regional heterogeneity across muscles (Kissane *et al*. 2018). In general, each region of interest of the soleus muscle contained ∼155 fibres and the diaphragm ∼70 fibres.

### *In silico* muscle PO_2_ modelling

Our model applied mathematical and computational frameworks to generate theoretical predictions of the cross‐sectional distribution of PO_2_ in the soleus and diaphragm using a custom MATLAB ‘oxygen transport modeller’, as previously described (Al‐Shammari *et al*. 2019). Briefly, using digitised images of muscle cryosections, individual fibre boundaries were identified, a phenotype assigned, and capillary locations defined. A computational framework was then established allowing a mathematical mesh of equations to be superimposed on realistic geometry. Tissue PO_2_ measurements were then derived by incorporating estimates (applied similarly in each group) of capillary radius (1.8−2.5 × 10^−4^ cm), muscle oxygen consumption (15.7 × 10^−5^ ml O_2_ ml^−1^ s^−1^), myoglobin concentration (10.2 × 10^−3^ ml O_2_ ml^−1^), O_2_ solubility (3.89 × 10^−5^ ml O_2_ ml^−1^ mmHg^−1^) and diffusivity (1.73 × 10^−7^ cm^2^ s^−1^) as detailed elsewhere (Al‐Shammari *et al*. 2019), with direct measurement of these specific parameters beyond the scope of the present study (Tickle *et al*. 2020). As such, any differences between groups in terms of mitochondrial function (or other assumed variables) were not accounted for in our model. Relevant biophysical parameters affecting O_2_ diffusion from reputable sources were used in the mathematical model to generate predictions of the cross‐sectional distribution of PO_2_ in a muscle biopsy under simulated resting and maximal oxygen consumption conditions. As with all biological models, inherent limitations prevent full characterisation of the wide myriad of interacting variables; however, relative changes within a given tissue were the key output and are likely robust. In line with former studies (Al‐Shammari *et al*. 2019), compensation for differences in many parameters, e.g. myoglobin saturation, have relatively small effects on the documented outcomes due to the dominant effect of capillary supply and fibre size on peripheral O_2_ transport.

### Statistical analyses

Following appropriate checks of normality, between‐group differences were assessed by unpaired two‐tailed Student's *t* tests. Contractile relationships were analysed as two‐way repeated measures ANOVA followed by the Bonferroni *post hoc* test, where appropriate. Analyses were performed in GraphPad Prism v.8. Data are presented as means ± SD, and the level of significance was set at *P* < 0.05 for all analyses.

## Results

### Cardiometabolic phenotype

**Figure 1:**
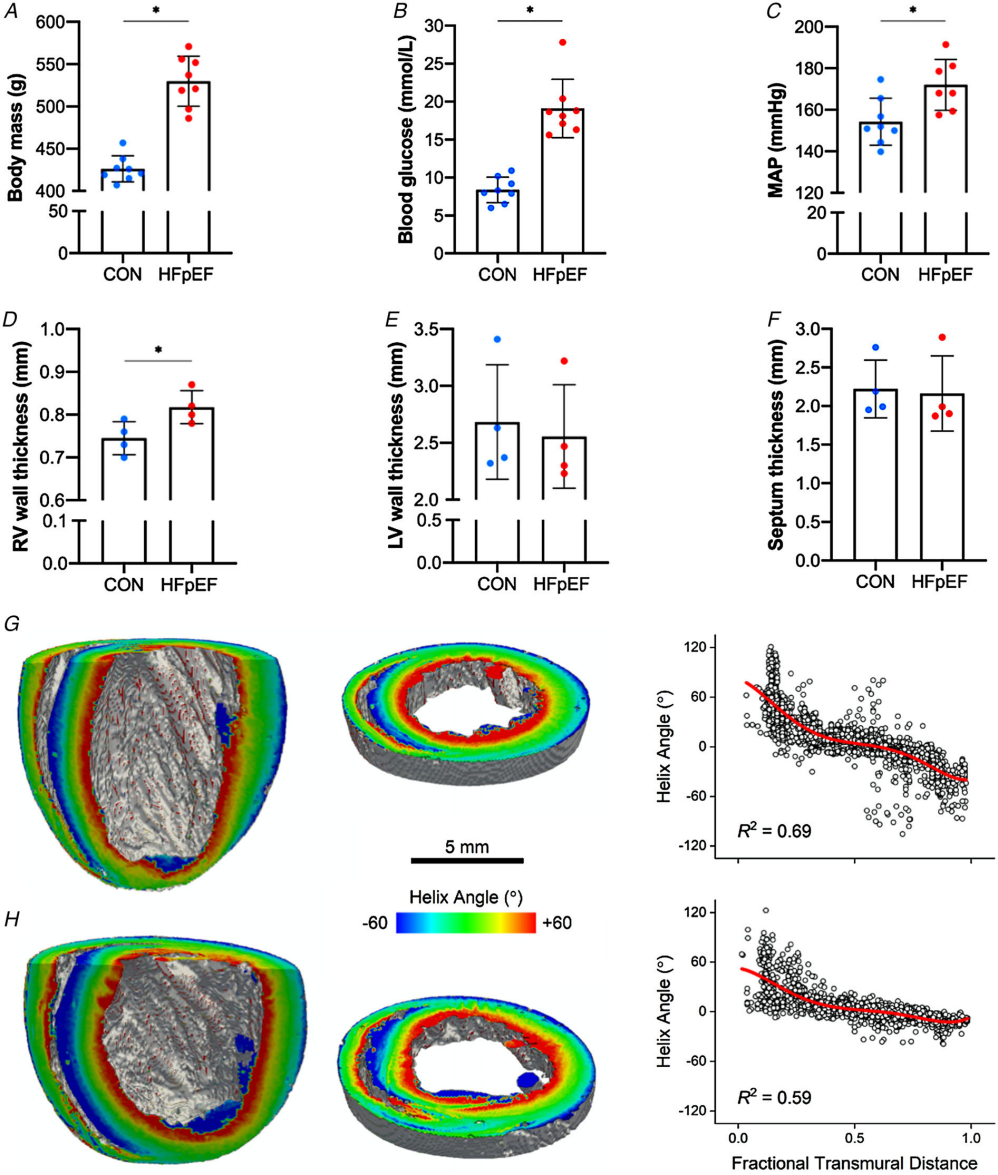
Cardiometabolic characteristics At 20 weeks of age, HFpEF rats developed obesity (426.25 ± 15.41 *vs*. 529.88 ± 29.56 g *P* < 0.001) (*A*), hyperglycaemia (8.38 ± 1.69 *vs*. 19.10 ± 3.83 mmol l^−1^; *P* < 0.001) (*B*) and hypertension (154.26 ± 11.28 *vs*. 172.03 ± 12.26 mmHg; *P = *0.012) (*C*). Compared with lean controls, obese‐HFpEF rats also showed increased right ventricular (RV) wall thickness (0.74 ± 0.04 *vs*. 0.82 ± 0.04 mm; *P = *0.034) (*D*); however, left ventricular (LV) wall and the septum thickness were not different between groups (2.68 ± 0.51 *vs*. 2.55 ± 0.46 mm; *P = *0.719 and 2.22 ± 0.38 *vs*. 2.16 ± 0.49 mm; *P* = 0.849, respectively) (*E–F*). Left and middle panels: long axis cuts (left) and short axis slices (middle) of representative lean (*G*) and obese (*H*) hearts, with myocyte helix (inclination) angle colour coded on the cut surfaces. Right panel: the helix angle in the RV free wall plotted as a function of fractional transmural distance (0.0, endocardium; 1.0, epicardium) for representative lean (*G*) and obese (*H*) hearts. The red continuous line is a 5th order polynomial fit to the data. Myocyte disarray is quantified by the *R* ^2^ of this fit. [Color figure can be viewed at wileyonlinelibrary.com]

As previously noted (Leite *et al*. 2015; Franssen *et al*. 2016; van Dijk *et al*. 2016; Bowen *et al*. 2017b; Schauer *et al*. 2020), by 20 weeks of age obese‐ZSF1 rats have developed typical metabolic signs associated with HFpEF including obesity (*P* < 0.001; Fig. 1 *A*), hyperglycaemia (*P* < 0.001; Fig. 1 *B*) and hypertension (*P *= 0.012; Fig. 1 *C*). In addition, obese rats developed cardiac remodelling typically associated with obese‐HFpEF that included right ventricular (RV) hypertrophy (*P *= 0.034; Fig. 1 *D*), although left ventricular (LV) and septal wall enlargement was not observed at this time point (*P *= 0.719; Fig. 1 *E* and *P *= 0.849; Fig. 1 *F*). Further, myocyte organisation/disarray was not significantly deteriorated either (RV: *P *= 0.971; LV: *P *= 0.13; septum: *P *= 0.064; Fig. 1 *G–H*).

### Histological and *in vitro* functional characteristics of the soleus muscle

**Figure 2:**
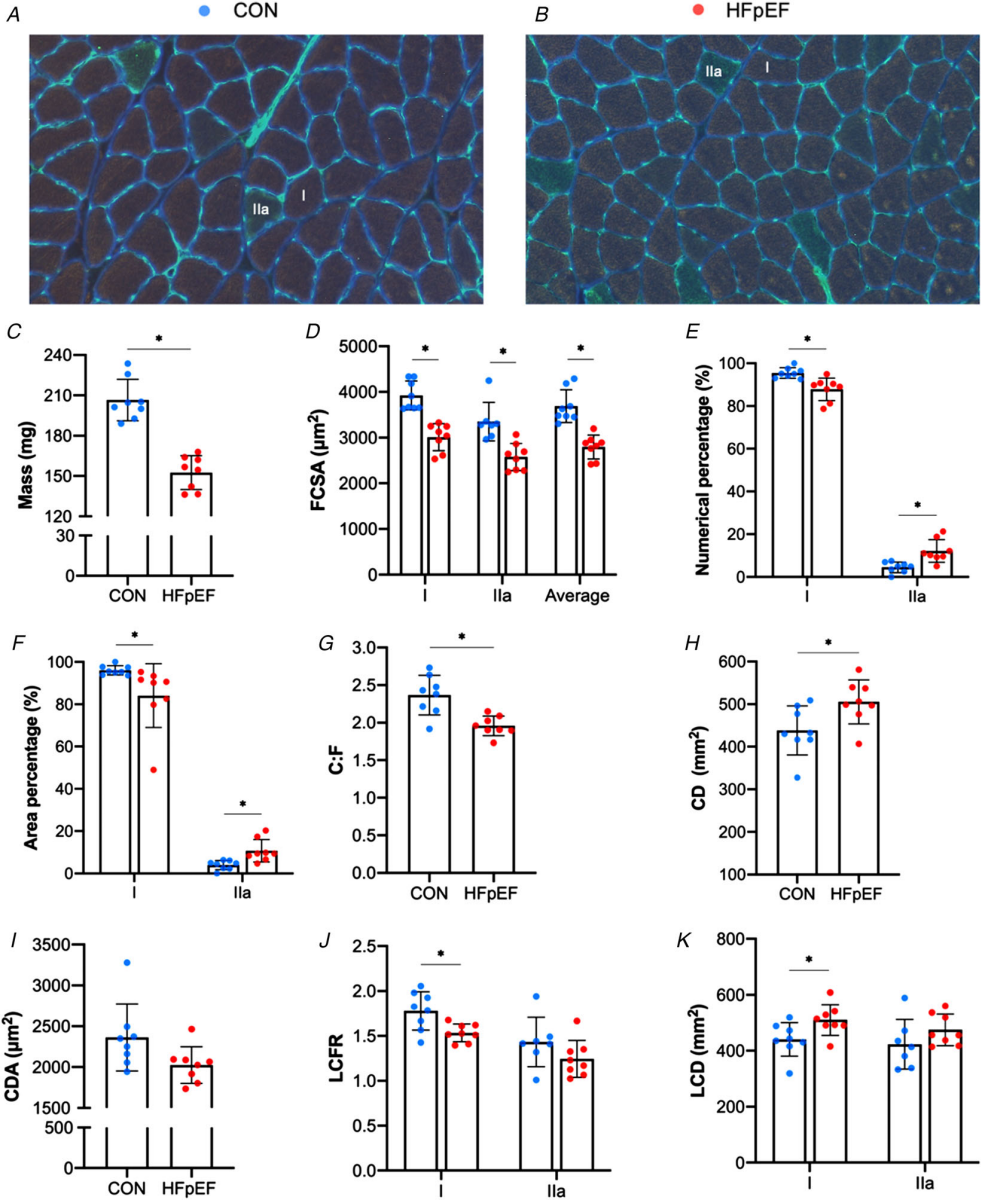
Histological features of the soleus muscle Representative soleus sections from control (*A*) and obese‐HFpEF (*B*). Obese‐HFpEF showed atrophy in the soleus muscle, with reduced wet muscle mass (206.54 ± 15.48 *vs*. 152.50 ± 12.55 mg; *P* < 0.001) (*C*) and reduced cross‐sectional area (CSA) in both Type I (3921.88 ± 316.51 *vs*. 3009.75 ± 298.03 μm^2^; *P* < 0.001) and Type IIa fibres (3351.43 ± 422.09 *vs*. 2575.69 ± 296.86 μm^2^; *P = *0.001) (*D*). HFpEF rats also had a lower numerical and areal composition of Type I fibres (95.49 ± 2.45 *vs*. 87.84 ± 5.32%, *P = *0.002 and 96.06 ± 2.17 *vs*. 84.06 ± 15.14 %, *P = *0.043, respectively), whereas these were higher in Type IIa fibres (4.51 ± 2.45 *vs*. 12.16 ± 5.32 %, *P = *0.002 and 3.94 ± 2.17 *vs*. 10.74 ± 5.31 %, *P = *0.005, respectively) (*E‐F*). Moreover, compared with lean controls, obese rats had reduced capillary‐to‐fibre (C:F) ratio (2.37 ± 0.26 *vs*. 1.96 ± 0.13; *P = *0.002) (*G*), whereas capillary density (CD), was increased (438.23 ± 57.66 *vs*. 505.5 ± 51.53 mm^−2^; *P = *0.027) (*H*) with no change in capillary domain area (CDA) (2363.28 ± 410.48 *vs*. 2024.48 ± 223.24 μm^2^; *P = *0.059) (*I*). Finally, local analyses of capillary distribution showed that HFpEF rats had lower local capillary‐to‐fibre ratio (LCFR) in Type I fibres (1.78 ± 0.22 *vs*. 1.53 ± 0.10; *P = *0.011), although this was unchanged in Type IIa fibres (1.43 ± 0.27 *vs*. 1.24 ± 0.21; *P = *0.154) (*J*). In contrast, local capillary density (LCD) in Type I fibres was increased in HFpEF rats (440.41 ± 59.71 *vs*. 510.15 ± 54.9 mm^−2^; *P = *0.029), with no changes in Type IIa fibres (423.20 ± 88.64 *vs*. 474.73 ± 56.17 mm^−2^; *P = *0.196) (*K*). [Color figure can be viewed at wileyonlinelibrary.com]

**Figure 3:**
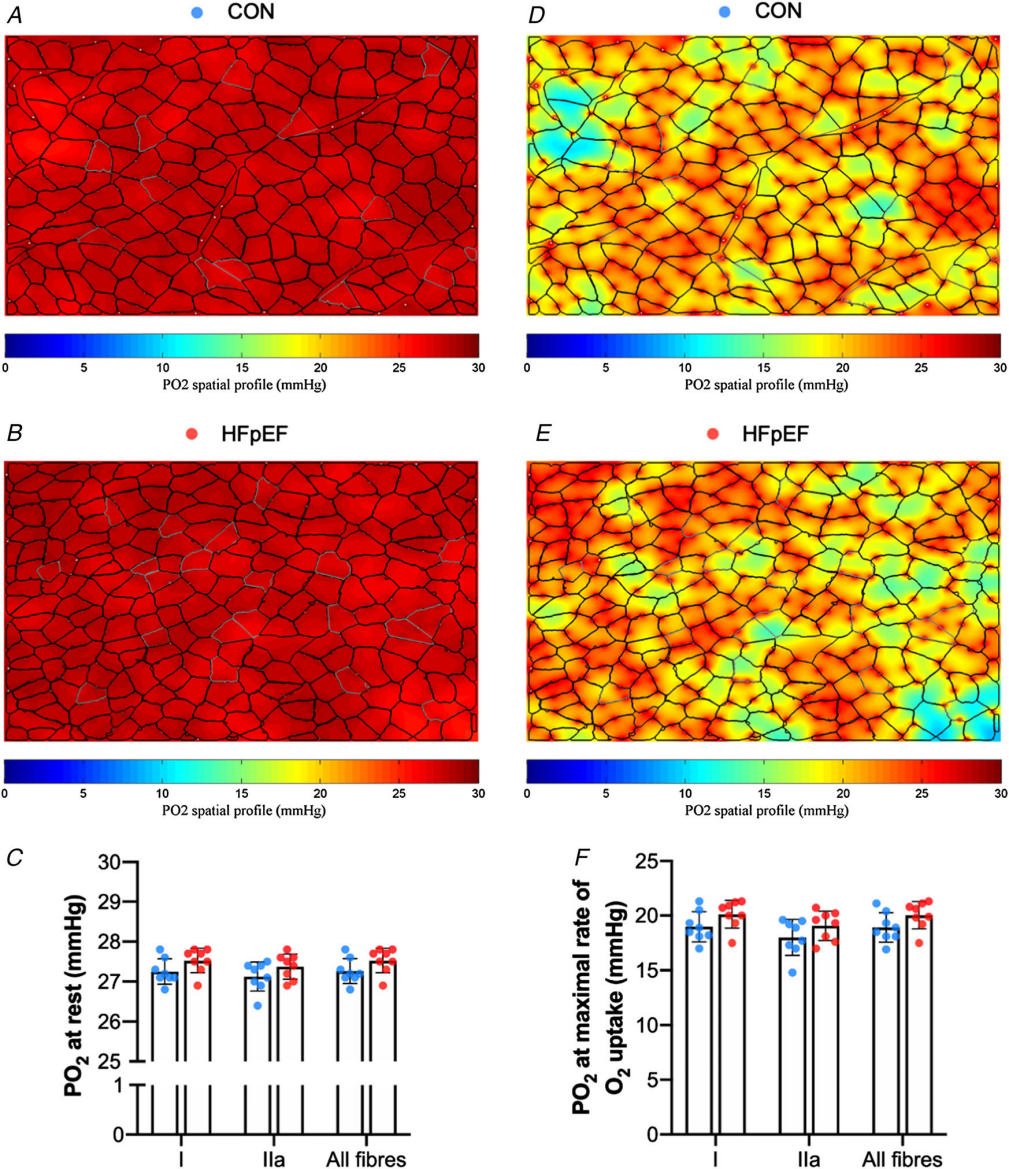
Modelling of soleus muscle oxygen tension Simulation of muscle PO_2_ at rest (*A–B*) and maximal rate of oxygen consumption (*D–E*) in representative images. There were no significant differences in simulations of muscle PO_2_ at rest (Type I fibres: 27.26 ± 0.33 *vs*. 27.54 ± 0.30 mmHg, *P = *0.099; Type IIa: 27.12 ± 0.39 *vs*. 27.37 ± 0.31 mmHg, *P = *0.16; all fibres: 27.25 ± 0.33 *vs*. 27.52 ± 0.29 mmHg, *P = *0.102) (*C*) or at maximal rate of oxygen consumption (Type I: 18.97 ± 1.38 *vs*. 20.11 ± 1.27 mmHg, *P = *0.109; Type IIa: 18.00 ± 1.64 *vs*. 19.07 ± 1.36 mmHg, *P = *0.177; all fibres: 18.92 ± 1.36 *vs*. 20.02 ± 1.24 mmHg, *P = *0.115) (*F*). Areas of muscle hypoxia (PO_2_ < 0.5 mmHg) are highlighted in blue. [Color figure can be viewed at wileyonlinelibrary.com]

As shown in representative muscle sections (Fig. 2 *A–B*), soleus from obese‐HFpEF rats demonstrated clear atrophy with a 26% lower wet mass (*P* < 0.001; Fig. 2 *C*) and a 23% lower CSA of both Type I (*P* < 0.001) and Type IIa fibres (*P *= 0.001; Fig. 2 *D*) when compared with lean controls. No Type IIx/b fibres were detected in either group. HFpEF rats also had a lower numerical and areal composition of Type I fibres (*P *= 0.002; Fig. 2 *E* and *P *= 0.043; Fig. 2 *F*, respectively), whereas these were higher in Type IIa fibres (*P *= 0.002 and *P *= 005, respectively). In addition, HFpEF rats had a lower C:F ratio (*P *= 0.002; Fig. 2 *G*) but a higher CD, indicating that atrophy proceeded at a greater rate than capillary loss (*P *= 0.027; Fig. 2 *H*), while CDA did not differ significantly (*P *= 0.059; Fig. 2 *I*). Analyses of local capillary distribution revealed that HFpEF rats had lower LCFR in Type I fibres (*P *= 0.011), and while a similar trend was found in Type IIa fibres this did not reach significance (*P *= 0.154; Fig. 2 *J*). In contrast, LCD in Type I fibres was higher in HFpEF rats (*P *= 0.029), and again while a similar trend was found in Type IIa fibres this did not reach significance (*P *= 0.196; Fig. 2 *K*). To understand whether HFpEF influenced muscle PO_2_, we simulated muscle oxygen tension (PO_2_) under resting (Fig. 3 *A–C*) and maximal demand (Fig. 3 *D–F*). No differences between groups were found after the calculation of muscle oxygenation at either rest (Type I fibres: *P *= 0.099; Type IIa: *P *= 0.167; all fibres: *P *= 0.102; Fig. 3 *C*) or maximal rate of oxygen consumption (Type I: *P *= 0.109; Type IIa: *P *= 0.177; all fibres: *P *= 0.115; Fig. 3 *F*).

**Figure 4:**
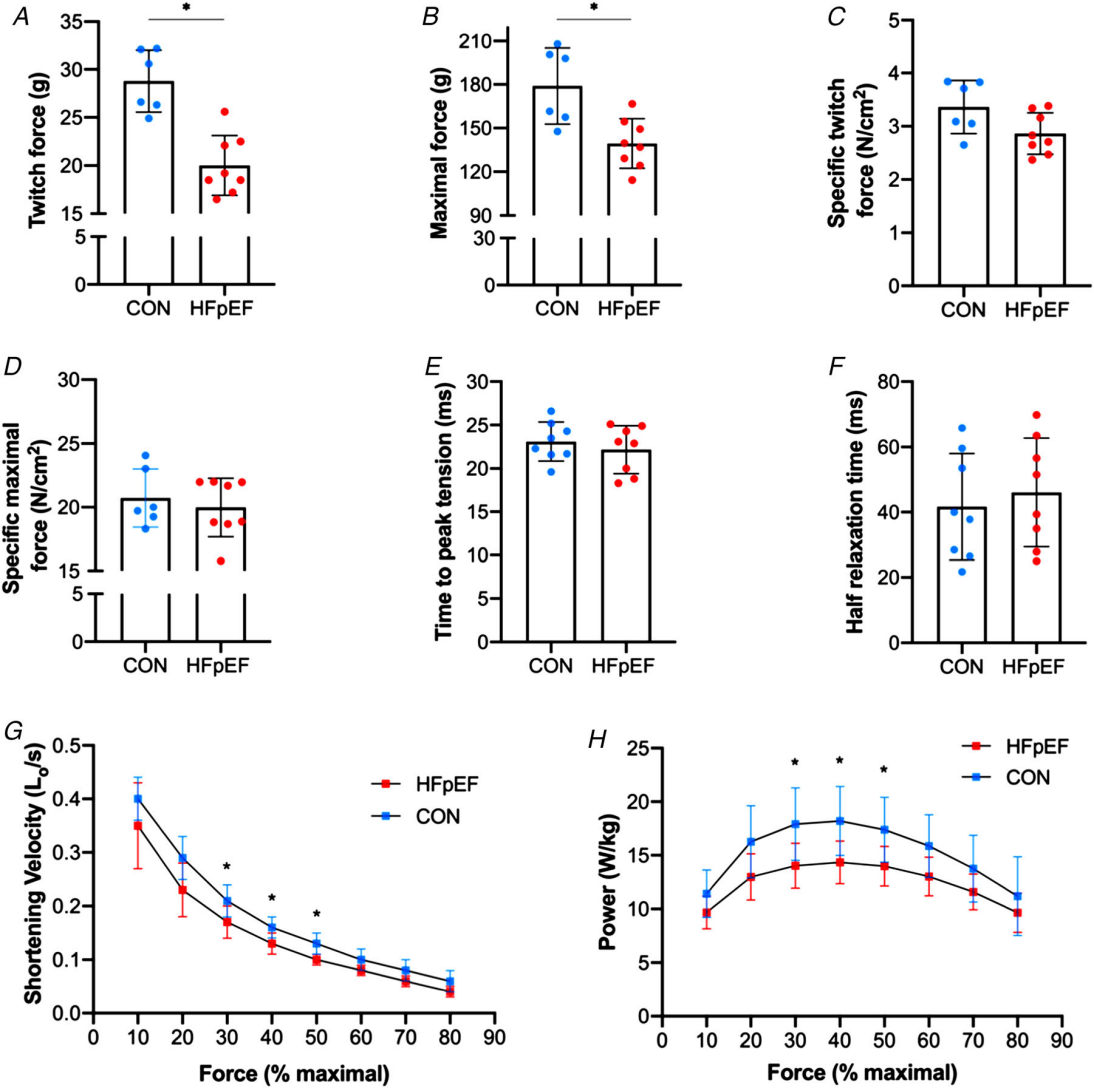
*In vitro* skeletal muscle function The soleus of HFpEF rats showed lower absolute twitch force (28.79 ± 3.23 *vs*. 20.01 ± 3.10 g; *P* < 0.001) (*A*) and absolute maximal tetanic force (178.95 ± 26.18 *vs*. 139.37 ± 17.05 g; *P = *0.005) (*B*), although mass‐specific twitch and maximal forces were similar between groups (3.36 ± 0.50 *vs*. 2.86 ± 0.39 N/cm^2^; *P = *0.056 and 20.72 ± 2.28 *vs*. 19.98 ± 2.29 N/cm^2^; *P = *0.557, respectively) (*C–D*). Similarly, time‐to‐peak tension and half relaxation time remained unchanged (23.10 ± 2.25 *vs*. 22.18 ± 2.75 ms; *P = *0.474 and 41.68 ± 16.33 *vs*. 46.08 ± 16.70 ms; *P = *0.603, respectively) (*E–F*). However, HFpEF rats showed impairments in shortening velocity and muscle power when measured across different percentages of their maximal force (30, 40 and 50%) (*P* < 0.05) (*G–H*). [Color figure can be viewed at wileyonlinelibrary.com]

The soleus generated lower absolute twitch and maximal forces in HFpEF than control rats (*P* < 0.001; Fig. 4 *A* and *P *= 0.005; Fig. 4 *B*, respectively), consistent with muscle atrophy, although after adjustment for muscle cross‐sectional area there was no difference between groups in specific forces (*P *= 0.056, Fig. 4 *C*; and *P *= 0.557, Fig. 4 *D*, respectively). Similarly, twitch characteristics of half relaxation time (*P *= 0.603; Fig. 4 *E*) and time‐to‐peak tension (*P *= 0.474; Fig. 4 *F*) remained unchanged, as did the maximal twitch:tetanus ratio between control and HFpEF (0.16 ± 0.03 *vs*. 0.14 ± 0.02; *P* = 0.140). However, HFpEF rats demonstrated impairments to both shortening velocity (range 10–17%) and mechanical power (range 14–22%) when measured across various percentages of their maximal force (*P* < 0.05; Fig. 4 *G–H*), suggesting obese‐HFpEF reduces intrinsic soleus contractile function related to muscle shortening rather than specific force. Interestingly, however, while *V*_max_ was not different between groups (1.16 ± 0.52 *vs*. 0.88 ± 0.33 *L*_0_/s; *P *= 0.307), there was a tendency for the force–velocity curvature to be 25% greater in HFpEF than controls (i.e. a lower power ratio: 0.06 ± 0.02 *vs*. 0.08 ± 0.02; *P *= 0.086).

### *In situ* muscle function and femoral artery blood flow

**Figure 5:**
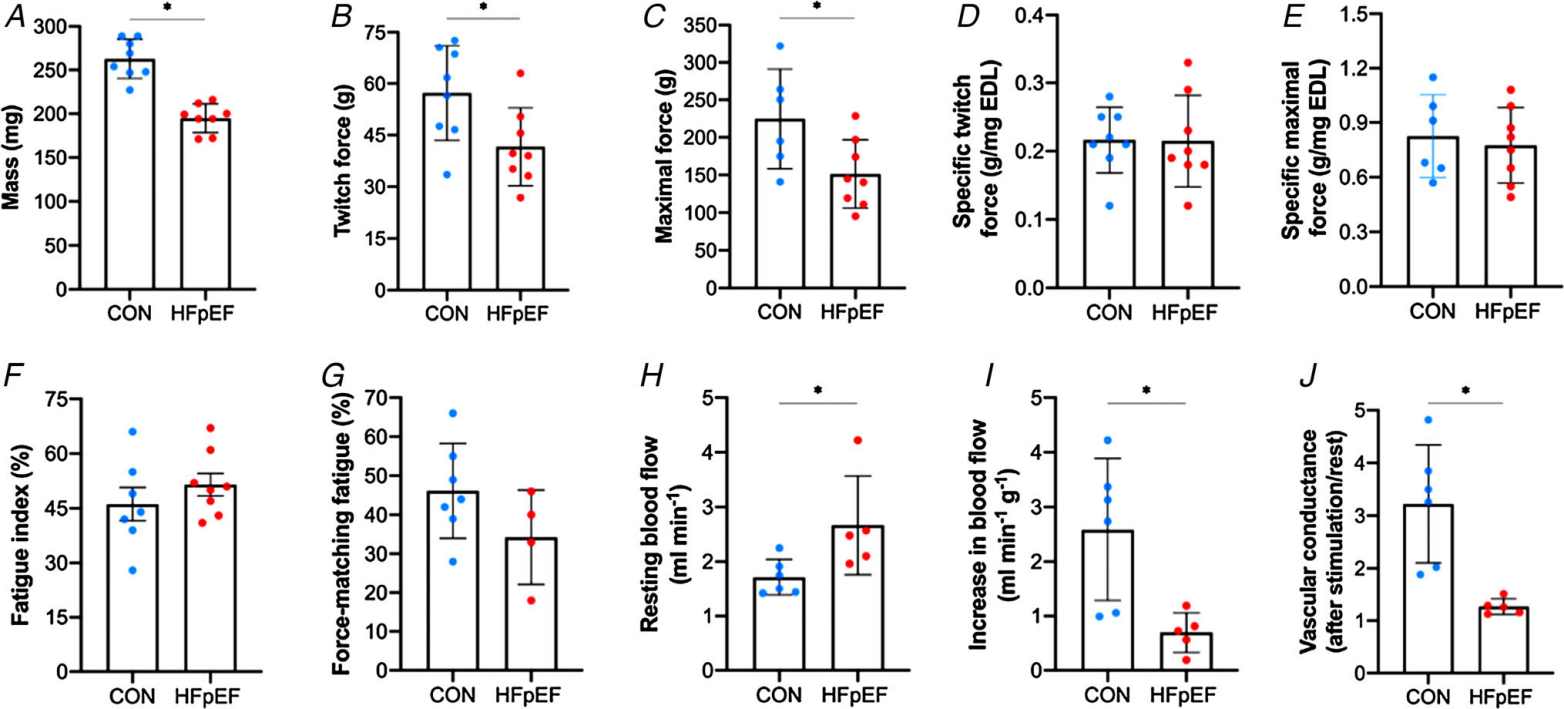
*In situ* EDL contractile function and femoral artery blood flow Absolute twitch and maximal tetanic forces of the EDL muscle were lower in HFpEF rats than in controls (57.24 ± 13.82 *vs*. 41.62 ± 11.31 g, *P = *0.016 and 224.64 ± 66.36 *vs*. 151.38 ± 45.45 g, *P = *0.030, respectively) (*B–C*). However, when normalised to muscle mass, which was reduced in HFpEF rats (262.88 ± 22.47 *vs*. 194.73 ± 15.35 mg; *P* < 0.001) (*A*), these were not significantly affected (0.22 ± 0.05 *vs*. 0.22 ± 0.07 g mg^−1^ EDL, *P* < 0.968 and 0.82 ± 0.23 *vs*. 0.77 ± 0.21 g mg^−1^ EDL, *P = *0.675, respectively) (*D–E*). The fatigue index was similar between groups (0.46 ± 0.12 *vs*. 0.51 ± 0.09 %; *P = *0.325) (*F*). However, HFpEF rats tended to be more fatigable during the force‐matched protocol (45.96 ± 11.96 *vs*. 34.35 ± 12.08; *P = *0.079) (*G*). Resting femoral artery blood flow was augmented in HFpEF rats (1.71 ± 0.33 *vs*. 2.66 ± 0.90 ml min^−1^; *P = *0.039) (*H*). In contrast, HFpEF rats showed an impaired increase in muscle‐specific EDL blood flow during stimulation (2.59 ± 1.30 *vs*. 0.69 ± 0.37 ml min^−1^ g^−1^; *P = *0.012) (*I*). Moreover, a reduction in the functional hyperaemic scope was also found in HFpEF (3.22 ± 1.12 *vs*. 1.27 ± 0.15; *P = *0.004) (*J*). [Color figure can be viewed at wileyonlinelibrary.com]

EDL muscle wet mass was 26% lower in HFpEF rats (*P* < 0.001; Fig. 5 *A*). This corresponded to lower absolute twitch and maximal forces of 27% and 33%, respectively (*P *= 0.016; Fig. 5 *B* and *P *= 0.030; Fig. 5 *C*, respectively). When normalised to muscle mass, twitch and maximal specific forces were not different between groups (*P *= 0.968; Fig. 5 *D* and *P *= 0.675; Fig. 5 *E*, respectively), while relative fatigability was unaffected (*P *= 0.325; Fig. 5 *F*). However, HFpEF rats tended to be around 26% more fatigable during the force‐matched protocol (*P *= 0.079; Fig. 5 *G*). While HFpEF rats had higher levels of resting femoral artery blood flow (*P *= 0.039; Fig. 5 *H*), they showed a severely blunted hyperaemic response of 73% to repeated contractions (*P* = 0.012; Fig. 5 *I*). Similarly, impairments in the hyperaemia calculated using vascular conductance was found in HFpEF (*P *= 0.004; Fig. 5 *J*). Overall, this suggests that while obese‐HFpEF does not induce muscle dysfunction related to neuromuscular transmission failure, a severe decrement to increase leg blood flow in response to contractions is apparent.

### Histological and functional characteristics of the diaphragm

**Figure 6:**
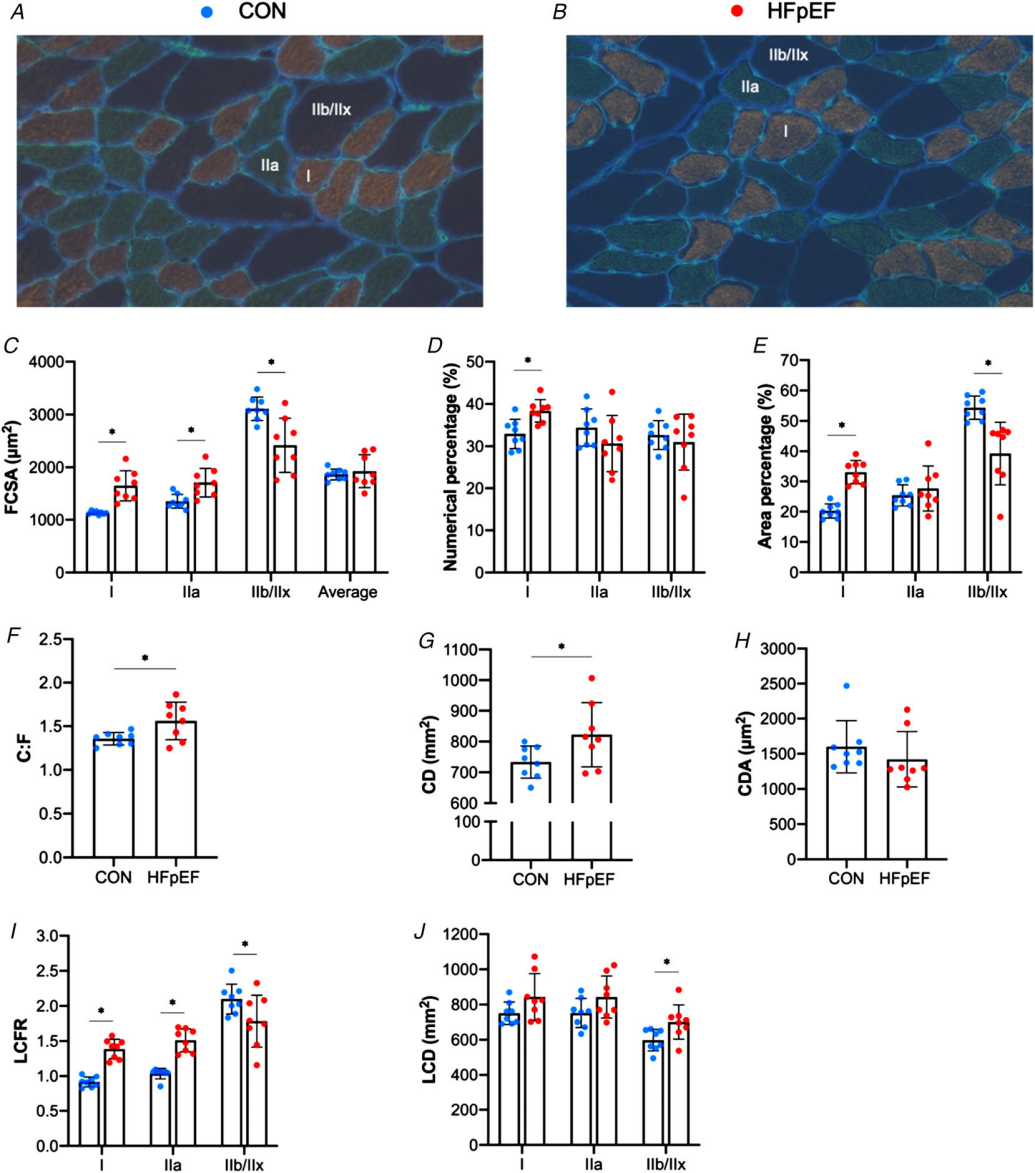
Histological features of the diaphragm Representative diaphragm sections from control (A) and obese‐HFpEF (B). Compared with lean controls, HFpEF rats had increased cross‐sectional area (CSA) in Type I (1130.67 ± 30.45 *vs*. 1647.83 ± 286.52 μm^2^; *P* < 0.001) and Type IIa fibres (1352.19 ± 133.46 *vs*. 1709.33 ± 273.24 μm^2^; *P = *0.005), whereas CSA of Type IIb/IIx fibres was reduced (3109.90 ± 222.49 *vs*. 2418.50 ± 514.36 μm^2^; *P = *0.004) (*C*). HFpEF rats also had a higher numerical percentage of Type I fibres (32.93 ± 3.46 *vs*. 38.38 ± 2.64 %; *P = *0.003), although this remained unchanged in Type IIa (34.41 ± 4.39 *vs*. 30.65 ± 6.67 %; *P = *0.203) and IIb/IIx fibres (32.63 ± 3.43 *vs*. 31.00 ± 6.63 %; *P = *0.545) (*D*). Additionally, HFpEF rats showed a higher area percentage of Type I fibres (20.26 ± 2.37 *vs*. 33.12 ± 3.85 %; *P* < 0.001), whereas this was unchanged in Type IIa fibres (25.38 ± 3.50 *vs*. 27.66 ± 7.47 %; *P = *0.449) and reduced in Type IIb/IIx fibres (54.36 ± 3.83 *vs*. 39.23 ± 10.33; *P = *0.002) (*E*). HFpEF rats also showed general and local alterations in capillary distribution. General changes included increased capillary‐to‐fibre (C:F) ratio (1.96 ± 0.12 *vs*. 2.26 ± 0.28; *P = *0.015) (*F*) and capillary density (CD) (733.42 ± 51.94 *vs*. 822.30 ± 104.43 mm^2^; *P = *0.049) (*G*), although capillary domain area (CDA) remained unchanged (1600.57 ± 371.08 *vs*. 1420.58 ± 392.25 μm^2^; *P = *0.362) (*H*). Local changes included increased LCFR in Type I (0.92 ± 0.08 *vs*. 1.39 ± 0.14; *P* < 0.001) and Type IIa fibres (1.05 ± 0.04 *vs*. 1.51 ± 0.16; *P* < 0.001) and reduced local capillary‐to‐fibre ratio (LCFR) in glycolytic/Type IIb/IIx fibres (2.13 ± 0.21 *vs*. 1.78 ± 0.37; *P = *0.040) (*I*). In contrast, however, HFpEF rats had increased local capillary density (LCD) in Type IIb/IIx fibres (615.85 ± 45.69 *vs*. 700.85 ± 97.37 mm^2^; *P = *0.042), with no changes in Type I (765.26 ± 60.76 *vs*. 843.27 ± 132.38 mm^2^; *P = *0.152) and Type IIa fibres (763.78 ± 68.71 *vs*. 842.75 ± 119.64 mm^2^; *P = *0.128) (*J*). [Color figure can be viewed at wileyonlinelibrary.com]

Representative diaphragm sections from control and HFpEF rats are presented in Fig. 6 *A–B*. Average FCSA was similar between groups (*P *= 0.609; Fig. 6 *C*). However, compared with lean controls HFpEF increased FCSA in both Type I and Type IIa fibres by 46% (*P* < 0.001) and 26% (*P *= 0.005), respectively (Fig. 6 *C*), but reduced Type IIb/IIx FCSA by 22% (*P *= 0.004). Additionally, HFpEF rats had a higher numerical percentage of Type I fibres (*P *= 0.003; Fig. 6 *D*) and a higher area percentage of Type I fibres (*P* < 0.001; Fig. 6 *E*).

**Figure 7:**
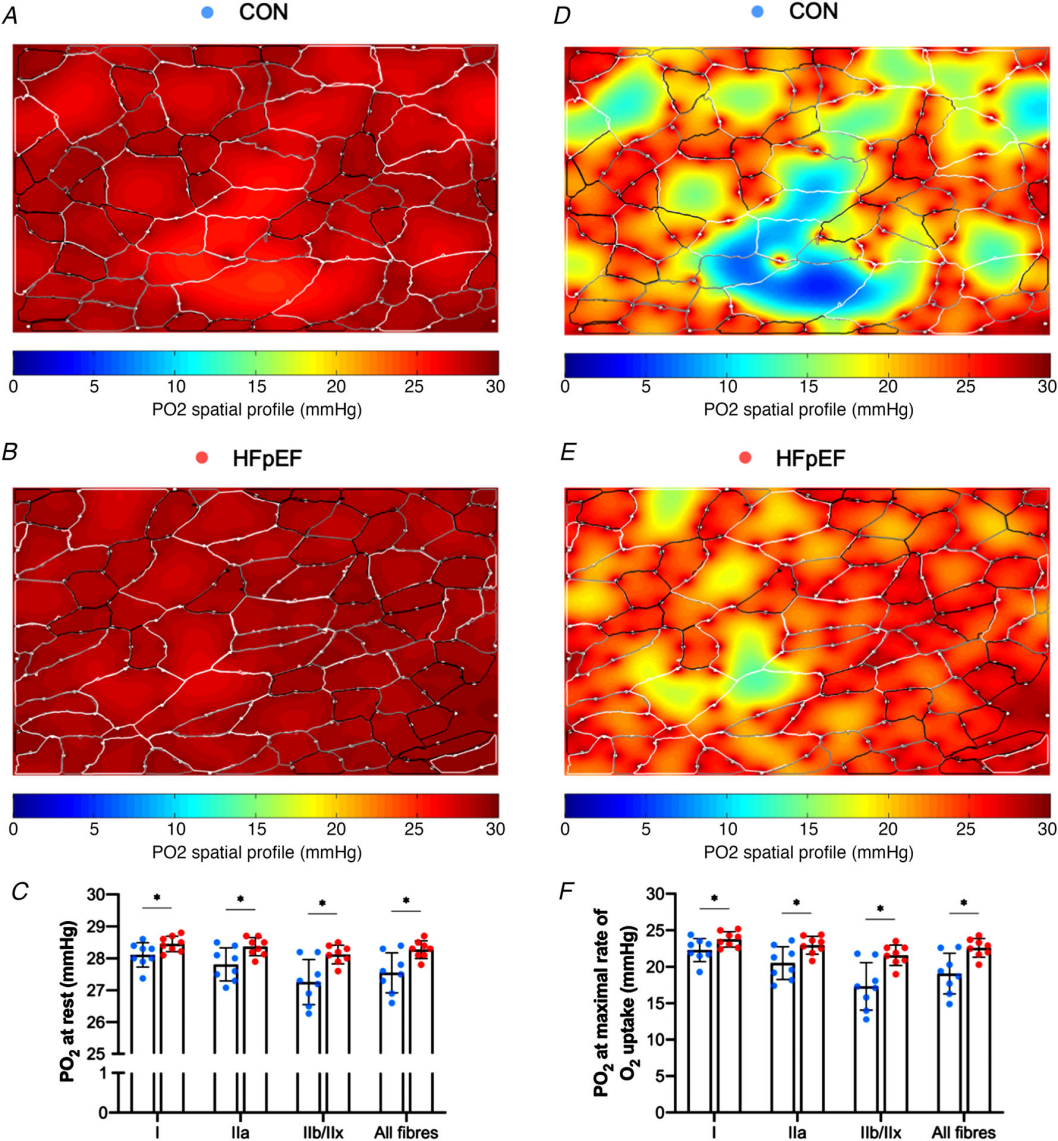
Modelling of diaphragm oxygen tension Simulation of muscle PO_2_ at rest (*A–B*) and maximal rate of oxygen consumption (*D–E*) in representative images. Compared with lean controls, HFpEF rats showed higher muscle oxygen tension at rest (Type I fibres: 28.09 ± 0.39 *vs*. 28.45 ± 0.24 mmHg, *P = *0.043; Type IIa: 27.80 ± 0.52 *vs*. 28.35 ± 0.27 mmHg, *P = *0.019; Type IIb/IIx: 27.26 ± 0.70 *vs*. 28.13 ± 0.29 mmHg, *P = *0.006; all fibres: 27.55 ± 0.61 *vs*. 28.28 ± 0.27, *P = *0.009) (*C*) or at maximal rate of oxygen consumption (Type I: 22.29 ± 1.57 *vs*. 23.76 ± 1.06 mmHg, *P = *0.045; Type IIa: 20.52 ± 2.27 *vs*. 22.95 ± 1.22 mmHg, *P = *0.018; Type IIb/IIx: 17.33 ± 3.24 *vs*. 21.58 ± 1.39 mmHg, *P = *0.004; all fibres: 19.07 ± 2.78 *vs*. 22.58 ± 1.26 mmHg, *P = *0.006) (*F*). [Color figure can be viewed at wileyonlinelibrary.com]

Global and local alterations were also observed in capillary distribution, with C:F ratio and CD increased in HFpEF rats (*P *= 0.015; Fig. 6 *F* and *P *= 0.049; Fig. 6 *G*, respectively) but CDA did not differ significantly (*P *= 0.362; Fig. 6 *H*). HFpEF rats had increased LCFR in Type I (*P* < 0.001) and Type IIa fibres (*P* < 0.001), whereas this was reduced in Type IIb/IIx fibres (*P *= 0.040; Fig. 6 *I*). In contrast, LCD was higher in HFpEF for Type IIb/IIx fibres (*P *= 0.042), with no changes in Type I (*P *= 0.152) and Type IIa fibres (*P *= 0.128; Fig. 6 *J*). We next estimated diaphragm PO_2_ levels (Fig. 7 *A–B*) and found HFpEF elevated resting muscle oxygen tension (Type I fibres: *P *= 0.043; Type IIa: *P *= 0.019; Type IIb/IIx: *P *= 0.006; all fibres: *P = *0.009; Fig. 7 *C*) and at maximal metabolic rates (Type I: *P *= 0.045; Type IIa: *P *= 0.018; Type IIb/IIx: *P *= 0.004; all fibres: *P *= 0.006; Fig. 7 *F*), indicating improved muscle oxygenation in obese‐HFpEF.

**Figure 8:**
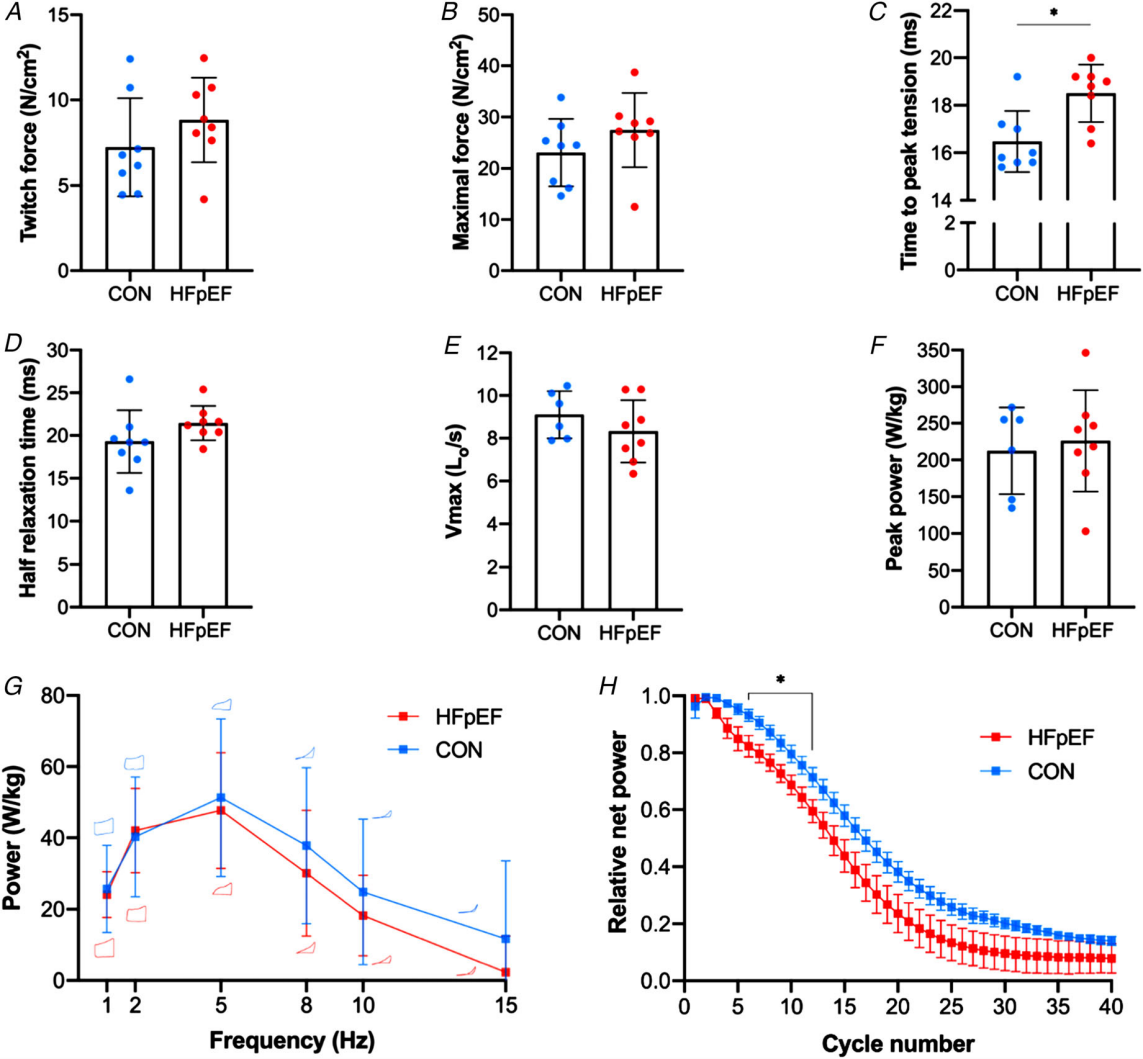
Functional properties of the diaphragm Isometric twitch and tetanic stress of the diaphragm were not different between groups (7.24 ± 2.87 *vs*. 8.83 ± 2.47 N/cm^2^, *P = *0.254 and 23.09 ± 6.56 *vs*. 27.46 ± 7.22 N/cm^2^, *P = *0.225, respectively) (*A–B*). In contrast, HFpEF rats showed slowed time‐to‐peak tension (16.48 ± 1.29 *vs*. 18.50 ± 1.21 ms; *P = *0.006) (*C*), although half relaxation time was not significantly affected (19.30 ± 3.68 *vs*. 21.45 ± 2.02 ms; *P = *0.170) (*D*). There were no differences in maximal shortening velocity (V_max_) (9.10 ± 1.11 *vs*. 8.32 ± 1.46 L_o_/s; *P = *0.278) (*E*) or peak isotonic power (212.56± 59.28 *vs*. 226.11 ± 69.36 W kg^−1^; *P = *0.701) (*F*) between groups. During cyclical contractions, while the net power‐cycle frequency relationship remained unaltered between groups (*P *> 0.05; typical work loops are shown at each cycle frequency for each group) (*G*), relative fatigue was greater in HFpEF (*P* < 0.001) under cycles 6–12 (*H*). [Color figure can be viewed at wileyonlinelibrary.com]

Isometric twitch and maximal tetanic stress of the diaphragm were similar between groups (*P *= 0.254; Fig. 8 *A* and *P *= 0.225; Fig. 8 *B*, respectively). However, analysis of twitch kinetics demonstrated that HFpEF rats had a slower time‐to‐peak tension (*P *= 0.006; Fig. 8 *C*) while half relaxation time remained unchanged (*P *= 0.170; Fig. 8 *D*) as did the maximal twitch:tetanus ratio between control and HFpEF (0.31 ± 0.06 *vs*. 0.32 ± 0.05; *P* = 0.630). Similarly, there were no differences in isotonic properties as assessed by maximal shortening velocity or maximal isotonic power between groups (*P *= 0.756; Fig. 8 *E* and *P *= 0.670; Fig. 8 *F*), with the power ratio also not different between groups (0.11 ± 0.01 *vs*. 0.11 ± 0.01; *P *= 0.253). During the cyclical contractions, there were no differences in the net power recorded at any given frequency and no difference in the cycle frequency that yielded maximum net power (i.e. 5 Hz for both groups) (all *P *> 0.05; Fig. 8 *G*). However, during repeated cyclical contractions the ability of the diaphragm to sustain work and power relative to the unfatigued state was reduced in HFpEF compared with controls (*P* = 0.001) and this occurred after relatively few cycles of work (cycles 6–12; *P* < 0.05; Fig. 8 *H*).

## Discussion

This study has identified novel skeletal muscle impairments in obese‐HFpEF that likely predispose towards the pathophysiology of exercise intolerance. The main findings from this study are:

1. Limb muscle weakness was closely associated with fibre atrophy in HFpEF, but isometric contractile properties were not impaired under neural‐ or direct‐muscle assessments, indicating preserved isometric neuromuscular function.
2. In contrast, limb isotonic muscle properties including shortening velocity and mechanical power were impaired in HFpEF.
3. An abnormal leg blood flow response to contractions alongside fibre type‐specific structural capillary loss was found in HFpEF, indicating perfusive O_2_ transport limitations.
4. Significant remodelling of the diaphragm occurred in HFpEF including divergent fibre‐type hypertrophy/atrophy, higher capillarity/PO_2_, and a Type II‐to‐I fibre‐type shift, with preserved muscle mechanics.

### Impact of HFpEF on limb muscle function

A reduction in skeletal muscle mass in patients with HFpEF is strongly associated with reduced muscle strength and poor quality of life (Bekfani *et al*. 2016). Muscle weakness is generally underpinned by either a reduction in muscle mass (i.e. atrophy) and/or intrinsic contractile dysfunction. In this study, we used *in vitro* (i.e. direct muscle) and *in situ* (i.e. peripheral nerve) stimulation approaches to assess isometric contractile properties in limb muscle. This allowed various sites in the muscle contractile process to be evaluated for dysfunction in HFpEF, including neuromuscular transmission and excitation–contraction coupling. Consistent with previous data where absolute maximal soleus force was reduced by ∼20% in HFpEF rats *vs*. controls (Bowen *et al*. 2018; Schauer *et al*. 2020), we observed that absolute twitch and maximal forces in both the soleus and EDL were lower in HFpEF rats. However, limb muscle weakness was closely associated with fibre atrophy as, after normalising for muscle mass, specific forces were not different between groups independent of whether the neural and blood supply remained intact. This is important, as it indicates that neuromuscular transmission and excitation–contraction coupling is likely preserved under isometric contractions in obese‐HFpEF.

However, most daily activities require the muscle to shorten against different loads to generate mechanical power and thus perform work. Therefore, assessment of muscle isotonic properties such as shortening velocity and power, which remained undefined in HFpEF, provides a more relevant assessment in relation to daily patient activities. Here, we observed that HFpEF rats had impairments to both shortening velocity and mechanical power in the soleus. This functional loss in HFpEF cannot be explained by a simple shift towards more Type I fibres (i.e. typically associated with slower shortening velocities where myosin heavy chain isoform is a key determinant (Bottinelli *et al*. 1991)), as we observed a higher proportion of Type IIa fibres in HFpEF (i.e., a Type I‐to‐II fibre‐type shift). As such, while this rules out a Type I fibre‐type shift as a potential mechanism underlying the slower shortening velocities observed in HFpEF, shortening velocity is also thought to be limited by the rate of ADP dissociation from actomyosin (Nyitrai *et al*. 2006). Thus, our data suggest obese‐HFpEF rats develop slowed rates of cross‐bridge detachment through impaired ADP release, potentially due to post‐translational modifications of myosin related to oxidative stress or glycation, as previously reported in HFrEF (Coirault *et al*. 2007) and ageing (Ramamurthy *et al*. 1999). In further support, slowed cross‐bridge kinetics have previously been reported in Type I and IIa fibres of vastus lateralis biopsies from patients with HFrEF (Miller *et al*. 2010), although other mechanisms such as impaired sarcoplasmic reticulum calcium pumping cannot be ruled out. Interestingly, we also found a tendency in the soleus for the curvature of the force–velocity relationship to be greater in HFpEF than the controls (i.e. where curvature is the inverse of the a/$P_{0}$ ratio in the Hill equation), which is in line with previous studies highlighting significant power loss during fatiguing exercise when the curvature is greater (or a/$P_{0}$ lower) (Jones, 2010). As such, a greater curvature of the force–velocity relationship may be another potential mechanism contributing to the loss of power observed in HFpEF. Overall, therefore, the significant loss of absolute limb force associated with muscle atrophy in obese‐HFpEF alongside intrinsic impairments related to lower shortening velocity and increased curvature would be predicted to severely reduce mechanical power and thus predispose towards exercise intolerance.

### Impact of HFpEF on limb skeletal muscle morphology

Despite skeletal muscle morphological alterations being well investigated in HFrEF (Kennel *et al*. 2015), little information is available on HFpEF from either animal or patient studies. Previous data from patients with HFpEF indicated the vastus lateralis Type I to Type II fibre‐type shift and a lower global C:F ratio, which is associated with reduced V̇O_2peak_ (Kitzman *et al*. 2014). Animal models (hypertensive or cardiometabolic) have shown in the soleus/EDL a significant fibre atrophy and a lower global C:F ratio (Bowen *et al*. 2015, 2017b, 2018; Schauer *et al*. 2020). Consistent with this, in the present study we found that soleus muscle from HFpEF rats exhibited a fibre atrophy of 24% and a Type IIa fibre‐type shift alongside a lower global C:F ratio of 17%. In contrast, however, we also provide new evidence that CD in the soleus was higher in HFpEF rats by 15% *vs*. controls, and by using novel local measures of capillarity (i.e. LCRF, LCD), we identified that while a similar trend was found in both fibre types, only Type I reached statistical significance. These additional local indices allowed us to further conclude that the lower C:F ratio observed in HFpEF was the result of a greater contribution from Type I fibres (i.e. LCRF), suggesting that slow‐ rather than fast‐twitch fibres tended to be more susceptible to microvascular alterations in this disease. However, the capillary supply per cross‐sectional area of Type I fibres (LCD) was in fact higher in HFpEF muscle. These global and local measures of capillarity where the C:F ratio was lower but CD higher in HFpEF are likely explained by the observed fibre atrophy, as CD is highly dependent upon fibre size (Egginton, 2011). This indicates that the degree of fibre atrophy exceeded the rate of capillary loss in HFpEF, thus increasing the CD, which has been suggested as an adaptive process to reduce diffusion distances across muscle fibres (Al‐Shammari *et al*. 2019), helping to discriminate structural from functional consequences of microvascular remodelling. A reduced distance for oxygen diffusion could be the result of an adaptation to changes in leg blood flow, as we observed. In HFpEF, this may also be a compensatory mechanism to preserve O_2_ flux from capillary to myocyte and thus maintain a better PO_2_ status across the muscle, not only in the face of capillary loss but also in response to the reported deficits in mitochondrial O_2_ utilisation (Bowen *et al*. 2015, 2017b; Molina *et al*. 2016).

In this regard, data collected from patients with HFpEF have identified clear impairments in the ability to widen arterial–venous O_2_ content (ΔAVO_2_) and augment peripheral oxygen extraction during exercise compared with HFrEF or controls (Haykowsky *et al*. 2011; Bhella *et al*. 2011a; Dhakal *et al*. 2015; Houstis *et al*. 2018; Zamani *et al*. 2020). It remains unclear whether the capillary loss we observed contributes to abnormal skeletal muscle O_2_ extraction at peak exercise in HFpEF, with a peripheral O_2_ diffusive limitation postulated as a major mechanism underpinning exercise intolerance in HFpEF (Dhakal *et al*. 2015; Houstis *et al*. 2018). To expand current knowledge (Dhakal *et al*. 2015; Houstis *et al*. 2018; Zamani *et al*. 2020), we therefore used *in silico* modelling to provide the first fibre‐type specific estimates of microvascular contribution to muscle oxygenation in HFpEF. We found muscle PO_2_ was similar between HFpEF and controls during simulated rest and at maximal exercise, which indicates adequate oxygenation is maintained in HFpEF with no evidence for enhanced tissue hypoxia. A recent patient study using forearm exercise found estimated peripheral O_2_ diffusion was not different between HFpEF and controls (Zamani *et al*. 2020), but this contrasts with previous cycling studies where O_2_ diffusion was significantly lower based on systemic haemodynamic, blood gas and pulmonary gas exchange measurements (Dhakal *et al*. 2015; Houstis *et al*. 2018). It should be noted that these patient studies did not measure leg/muscle ΔAVO_2_, fibre size, capillarity, or microvascular distribution, which can all influence peripheral O_2_ diffusion. While the current data do not allow us to directly confirm whether O_2_ diffusion was impaired in obese‐HFpEF, our data suggest that fibre morphology and capillary distribution are unlikely to contribute to O_2_ diffusive limitations.

Interestingly, and in contrast to HFrEF or controls, patients with HFpEF are unable to lower venous PO_2_ during exercise and therefore demonstrate a blunted peripheral O_2_ extraction response (Dhakal *et al*. 2015; Houstis *et al*. 2018; Poole *et al*. 2018; Zamani *et al*. 2020). The extent of this impaired muscle O_2_ extraction in HFpEF is likely explained, at least in part, by the significant mitochondrial abnormalities reported in patients with HFpEF (Molina *et al*. 2016). In our study, estimated muscle PO_2_ in HFpEF was not lower than controls at simulated maximal exercise, and while not discounting the potential for a muscle O_2_ diffusion limitation (Poole *et al*. 2018), this is consistent with maintenance of a high muscle oxygenation across the muscle in HFpEF to support more optimally functioning mitochondria (Al‐Shammari *et al*. 2019). Clearly, more studies are warranted to clarify the role of limitations to muscle O_2_ diffusion in HFpEF. However, our present data of a reduced leg blood flow (see below) implicate a perfusive O_2_ delivery limitation as one key mechanism that could potentially blunt O_2_ extraction in obese‐HFpEF.

### Impaired muscle blood flow response in HFpEF

Up until now, direct measures of leg (muscle) arterial blood flow had not been assessed in HFpEF, limiting mechanistic understanding. In the present study, using perivascular flow probes (the gold standard for measuring volumetric blood flow in animal studies), we directly demonstrated that the functional hyperaemic response to contractions was blunted in HFpEF rats. These data support the concept that the peripheral response to exercise is impaired in HFpEF (Houstis *et al*. 2018), and are consistent with non‐invasive measurements in patients with HFpEF during knee‐extensor exercise where impaired leg blood flow and vascular conductance occurred independently of limitations to heart rate, stroke volume and cardiac output (Lee *et al*. 2016b; Weavil *et al*. 2020) but contrast with recent isometric forearm data which did not find any change (Zamani *et al*. 2020). However, some (Maréchaux *et al*. 2016; Lee *et al*. 2016b; Kishimoto *et al*. 2017; Weavil *et al*. 2020) but not others (Hundley *et al*. 2007; Haykowsky *et al*. 2013; Lee *et al*. 2016a; Zamani *et al*. 2020) report an abnormal blood flood response to exercise in HFpEF patients when compared with controls, which is probably related to differences in the muscle studied, non‐invasive and different measurement techniques, and patient heterogeneity. Overall, our data support the potential for perfusive, feed‐artery O_2_ transport limitations in HFpEF, which likely contributes to exercise intolerance in this disease (Poole *et al*. 2018; Weavil *et al*. 2020).

Although the mechanisms underlying abnormal limb blood flow response to exercise in HFpEF remain unclear, endothelial function is impaired (Schmederer *et al*. 2018) and postulated as a central mechanism underlying disease progression (Paulus & Tschöpe, 2013; Gevaert *et al*. 2017; Schmederer *et al*. 2018). Beyond this, upstream central impairments related to cardiac output likely play a major role (Wolsk *et al*. 2019). While the functional significance of an impairment to limb blood flow during exercise in HFpEF is not known, it may exacerbate the degree of muscle fatigue experienced and limit daily activities performed by patients where the ability to repeatedly sustain absolute forces rather than relative forces becomes crucial, as recently demonstrated in patients with HFpEF (Weavil *et al*. 2020). This is supported by our employed matched‐initial force fatigue protocol, where HFpEF rats showed a trend to be 26% more fatigable than controls. These data provide important clinical relevance, as patients with muscle weakness such as HFpEF are often required to increase motor firing frequencies to perform certain daily activities that induce early fatigue (Ferreira *et al*. 2010; Weavil *et al*. 2020), although it is important to note this may not necessarily reflect direct differences in relation to muscle fatigue properties. Our data therefore indicate that skeletal muscle arterial blood delivery is potentially constrained during exercise in HFpEF. As such, at least in the context of this animal model and in support of recent patient findings (Weavil *et al*. 2020), a perfusive O_2_ delivery limitation could play a key role limiting O_2_ extraction and thus exacerbating exercise intolerance in obese‐HFpEF (Poole *et al*. 2018; Zamani *et al*. 2020). Interestingly, a lower leg blood flow during contractions coupled with a higher muscle CD in obese‐HFpEF could result in an increased red blood cell transit time to mediate a greater O_2_ extraction. However, this may not transpire in HFpEF due to the significant mitochondrial abnormalities developed in patients (Molina *et al*. 2016), thus preventing any significant widening of the ΔAVO_2_.

### Impact of HFpEF on diaphragm remodelling and muscle mechanics

Inspiratory (i.e. diaphragm) muscle weakness is evident and closely associated with symptoms of dyspnoea and poor prognosis in patients with HFpEF (Lavietes *et al*. 2004; Hamazaki *et al*. 2020). Multiple alterations to the diaphragm have been reported in HFpEF, including *in vitro* muscle weakness and fatigue alongside a Type II‐to‐I fibre‐type shift, fibre atrophy, and impaired *in situ* mitochondrial respiration in a hypertensive rat model (Bowen *et al*. 2015). In contrast, we show remodelling of the HFpEF diaphragm that is reminiscent of exercise‐training including fibre hypertrophy, increased mitochondrial content, and preserved fatigue resistance, although evidence for mitochondrial uncoupling and a mild isometric contractile dysfunction have been noted (Bowen *et al*. 2017b). The disparity in findings between models is likely explained by the co‐morbidity of obesity and its associated chronic respiratory loading, which can act as a training stimulus to increase fibre size, mitochondrial function/content, and fatigue resistance (Farkas *et al*. 1994; Powers *et al*. 1996). Whether similar findings are observed between obese *vs*. lean patients with HFpEF remains unknown.

Given that approximately 80% of HFpEF patients are obese (Shah *et al*. 2016) and a recent distinct obese‐HFpEF phenotype has been established (Obokata *et al*. 2017), the present study contributes novel and highly relevant data in relation to diaphragm plasticity. Until now, only limited data have been available with respect to fibre‐type structure, isoform, and microvasculature of the diaphragm in HFpEF (Bowen *et al*. 2015, 2017b). In the present study, we identified three major fibre types (Types I, IIa and IIb/IIx) to provide new evidence of a divergent hypertrophy/atrophy fibre remodelling in obese‐HFpEF alongside increased indices of global and local capillarity (i.e. C:F ratio and CD), and estimated levels of fibre oxygenation at rest and maximal exercise. Specifically, compared with controls, obese‐HFpEF rats had increased Type I/IIa FCSA, and reduced Type IIb/IIx FCSA, indicating compensatory adaptations in slow‐twitch fibres. This observation corresponds to the morphometric alterations following unilateral denervation of rat diaphragm muscle, where hypertrophy in Type I fibres but atrophy in Type IIb/IIx fibres occurred (Aravamudan *et al*. 2006). In support, albeit in the limb gastrocnemius, denervation has been demonstrated to induce fibre atrophy at a relatively higher rate than capillary rarefaction to mediate a higher CD (Paudyal *et al*. 2018), which is in line with our findings from the diaphragm in HFpEF. Nevertheless, it remains unclear whether obese‐HFpEF induces partial diaphragm muscle denervation in fast‐twitch Type IIb/x as reported in other conditions such as ageing (Elliott *et al*. 2016).

Overall the obese‐HFpEF diaphragm demonstrates improved indices of oxygen transport (increased capillarity and PO_2_ distribution) that likely supports the observed shift towards an oxidative phenotype (i.e. higher proportion of Type I fibres, mitochondrial content, and antioxidative enzyme capacity (Bowen *et al*. 2015, 2017b). This suggests that any functional diaphragm impairments developed in obese‐HFpEF were likely offset by morphological adaptations. To decipher this potential trade‐off, we performed *in vitro* isometric, isotonic and cyclical contractions on the diaphragm in one of the most detailed functional assessments in HFpEF to date. Our data indicate that impaired muscle mechanics and intrinsic diaphragm dysfunction generally do not develop early in the time course of obese‐HFpEF (∼20 weeks), with only a mild increase found compared with controls in terms of fatigability. Again, these data conflict with previous experimental data where a significant reduction in diaphragm force during repeated isometric contractions was measured in rats with more advanced hypertensive‐induced HFpEF (Bowen *et al*. 2015). In the present study we also simulated *in vivo* respiratory muscle mechanics, by applying cyclical length changes and phasic stimulation to the muscle to generate cycles of work, using the *in vitro* work loop technique (Josephson, 1985). Similar to our isolated isometric and isotonic measures, net power output during unfatigued cyclical contractions in obese‐HFpEF was unaffected in the diaphragm, but we did observe a mild increase in fatigue during repeated cyclical contractions. Collectively, these data suggest that respiratory muscle dysfunction is unlikely to be a key player in the pathogenesis of exercise intolerance in obese‐HFpEF, at least during early disease progression.

### Study limitations

We did not use echocardiography or invasive haemodynamics to quantify the extent of LV diastolic function and ejection fraction. However, this rat model has been validated and consistently develops key features of HFpEF as early as 10–15 weeks of age (Schauer *et al*. 2020), including impaired diastolic function, preserved ejection fraction, myocardial remodelling, and exercise intolerance (Hamdani *et al*. 2013; Leite *et al*. 2015; Franssen *et al*. 2016; van Dijk *et al*. 2016; Bowen *et al*. 2017b). Instead, we used MRI to confirm the presence of cardiac remodelling which occurred in the RV and this is known to be closely associated with HFpEF development in obese patients (Obokata *et al*. 2017) and one of the strongest predictors of poor prognosis (Burke *et al*. 2014). We also compared groups at a relatively early time point in the progression of HFpEF, which may limit translation of our findings to more advanced stages of the disease. In addition, our experiments were performed in male rats only such that it remains unclear whether similar findings would be observed in females, although a recent study confirmed that a similar time course in disease progression is observed in females (Schauer *et al*. 2020). Further, while differences in physical activity levels between groups were not measured and cannot be ruled out as having an influence on our experimental measures, it is well established that disuse alone fails to account for the skeletal muscle impairments developed during heart failure (Simonini *et al*. 1996; Miller *et al*. 2009). While we saw a trend for fatigue to be higher in HFpEF under our matched‐initial force protocol, this was only performed in *n *= 4 and thus may have been underpowered to detect statistical, rather than biologically meaningful, differences. Furthermore, as with all biological models, inherent limitations should be considered and our estimated muscle PO_2_ values from this study were based on a number of assumptions (as detailed in *Methods*) and may not be directly comparable to humans due to allometric scaling and/or fibre‐type differences.

## Conclusions

Obese‐HFpEF rats have a blunted hindlimb blood flow response to contractions alongside microvascular structural remodelling, fibre atrophy, and isotonic contractile dysfunction, which may be important factors underlying exercise intolerance in this disease. In contrast, diaphragm phenotype was largely preserved, indicating a more prominent role for limb rather than respiratory muscle abnormalities in obese‐HFpEF.

## Additional information

### Competing interests

None declared.

### Author contributions

E.E.G. performed the histological staining and image analyses and drafted the manuscript. P.G.T. performed *in situ* experiments and helped draft the manuscript. G.N.A. performed the *in vitro* diaphragm experiments and helped draft the manuscript. R.K. performed the *in vitro* diaphragm experiments and helped draft the manuscript. A.P.B. performed the cardiac imaging experiments and helped draft the manuscript. S.E. contributed to conception and design of the experiments, and interpretation of the data. T.S.B. contributed to conception and design of the study, performed muscle experiments, helped interpret data and draft the manuscript. All authors approved the final version of the manuscript.

### Funding

E.E.G. is a recipient of the Doctoral Fellowship from the Mexican National Council of Science and Technology (CONACYT). A.P.B. was supported by British Heart Foundation grant PG/16/74/32374. T.S.B. is a recipient of a Medical Research Council (MRC) New Investigator Research Grant (NIRG) (MR/S025472/1) and also received a Research Grant from The Physiological Society to support this work.

## Supporting information


**Statistical Summary Document**

## Data Availability

The data that support the findings of this study are available from the corresponding author upon reasonable request.
